# I Want Our Relationship to Last: Strategies That People Employ in Order to Improve Their Intimate Relationships

**DOI:** 10.1177/14747049221147154

**Published:** 2022-12-25

**Authors:** Menelaos Apostolou, Maria Argyridou, Eirini Evaggelia Nikoloudi, Timo Juhani Lajunen

**Affiliations:** 1 Department of Social Sciences, 121343 University of Nicosia, Nicosia, Cyprus; 2 Department of Psychology, 8018 Norwegian University of Science and Technology (NTNU), Trondheim, Norway

**Keywords:** improving relationships, relationship enhancement strategies, relationship difficulties, mating strategies, mating

## Abstract

Intimate relationships are not always easy to keep. Accordingly, the current research has attempted to identify the strategies that people employ in order to improve their relationships with their partners. In particular, by using open-ended questionnaires in a sample of 219 Greek-speaking participants, we have identified 81 acts that people were likely to perform toward this end. Subsequently, using quantitative research methods in a sample of 511 Greek-speaking participants, we classified these acts into 14 broader relationship enhancement strategies. Trying to understand partners' needs, discussing problems with partners and showing more interest in a partner, were among the most likely to be used strategies. Moreover, we asked a sample of 367 Greek-speaking participants to rate how effective these strategies would be on them, and we found that, trying to understand partners' needs, discussing problems with partners, and showing one's love to a partner, were rated as the most effective strategies. Men and women, and people of different ages, were generally in agreement over what strategies they were willing to use, and how effective these strategies would be on improving their relationship with their partners.

## Introduction

Keeping an intimate relationship is not an easy task. For instance, it has been estimated that about one in two marriages is likely to fail (Cherlin, 2009; Raley & Bumpass, 2003). In a recent study, about one-third of Greek- and Chinese-speaking participants said that they faced difficulties in keeping an intimate relationship (Apostolou & Wang, 2019). Despite these difficulties, people are motivated to keep and improve their intimate relationships (Apostolou, 2022; Baumeister & Leary, 1995), which raises the question of how they do so that the current research was designed to answer. In particular, the present study aimed to identify the strategies that people employ in order to improve their intimate relationships as well as to assess their effectiveness in achieving this goal.

### Strategies for Improving Intimate Relationships

#### Mate Retention Strategies

Partner defection leads to the loss of a valuable reproductive resource, as well as forfeiting all the investment diverted in attracting and retaining the intimate partner. It follows that partner defection would lead to a considerable decrease in one's reproductive success or fitness. This fitness loss would result in the evolution of strategies that would decrease the probability of losing their partners to others (Buss, 1988; Buss & Shackelford, 1997). Mate retention strategies comprise a broad range of behaviors ranging from acts of kindness and resource provisioning to vigilance, manipulation, and violence (Albert & Arnocky, 2016). These strategies can conceptually be divided into two categories, namely cost-inflicting behaviors and benefit provision behaviors (Buss, 1988). Cost-inflicting mate retention behaviors aim to decrease the probability of defection by reducing partners' self-esteem, and make them feel unworthy of the current relationship or by inducing fear and social isolation. On the other hand, benefit-provisioning mate retention behaviors reduce the likelihood of partner defection by enhancing the partner's satisfaction with the relationship (Albert & Arnocky, 2016; Buss, 1988). The current research aimed to identify benefit-provisioning mate retention behaviors and classify them into broader strategies that people use in order to improve their relationships. In order to understand these strategies, we need to understand first what causes relationship strain.

#### Sources of Relationship Strain

Most human evolution took place in a pre-industrial context, where our ancestors lived as hunters and gatherers or agropastoralists (Tooby & Cosmides, 2015). In such context, there were no social support institutions, and people had to rely on the assistance of others in order to care for themselves and their offspring. Accordingly, having an intimate partner to provide reliable support and assistance would make a considerable difference to one's survival and reproductive success or fitness. Such assistance was even more important if one considers that children require considerable investment for a long period before they become independent from their parents (Lancaster & Lancaster, 1987). In effect, especially in the pre-industrial context, children who receive support from both parents have better chances of survival than children who receive support from one (Hawkes et al., 1989; Kim et al., 2012). These factors have favored the evolution of a long-term mating strategy, where people strive to form long-term intimate relationships (Buss & Schmitt, 1993). We use here the term strategy to denote a set of actions motivated by evolved mechanisms that aim to achieve a specific goal, which in the present case, is to attract and keep a partner in the long run (see also Gangestad & Simpson, 2000). For this purpose, they look for partners who have desirable traits such as good resource provision capacity and good character that would make them good long-term mates (Buss & Schmitt, 2019; Thomas et al., 2020; Walter et al., 2020).

In this argument, people form intimate relationships in order to have around them individuals who could provide reliable and long-term support to them and to their current or prospective children. It follows that relationship strain would arise when partners behave in a way that indicates lack of commitment to the relationship; for instance, they neglect their mates, do not devote time to them, flirt with others, and so on. Similarly, relationship strain would arise from partners demonstrating lack of qualities such as a good resource provision capacity or a good character (Apostolou & Wang, 2020, 2021).

Moving on, another source of relationship strain is the adoption of an infidelity strategy. More specifically, extra-pair mating has potentially several benefits. For instance, it could enable men to increase their reproductive success by gaining sexual access to more partners, women to secure resources from extra-pair partners, and both men and women to probe other partners in case their current ones abandon them or die (Buss, 2000; Buss et al., 2017). Such benefits have favored the evolution of an infidelity strategy, where people form long-term intimate relationships as well as casual extra-pair ones (Buss & Schmitt, 1993). Yet, extra-pair mating can have considerable negative consequences for the legitimate partner, including losing the partner to another individual, raising other men's children, contracting a sexually transmitted disease, and losing part of a partner's investment to others (Buss, 2016).

These potential costs have favored the evolution of mechanisms such as jealousy that would enable people to protect themselves from infidelity (Buss, 2000). These mechanisms work on the basis of the smoke detector principle (Nesse, 2005, 2018). More specifically, smoke detectors are not perfect, and thus, they may not detect a fire when there is one (false negative), or they may detect a fire when there is not one (false positive). The first error may lead to death, while the second to a harmless nuisance. Accordingly, smoke detectors are manufactured to minimize false negatives by being too sensitive to cues suggesting that there is one, producing more false positives. In the same vein, a partner's infidelity can have detrimental consequences for one's fitness, so infidelity detection mechanisms may have been selected to produce more false positives in order to minimize false negatives. That is, they may frequently trigger, even in the absence of reliable cues of infidelity. Doing so, however, may tax the relationship, as it can result in many instances of partners accusing each other for things they did not do. Overall, it can be argued that a partner exhibiting lack of commitment, lacking desirable traits, and being controlling, constitute the main sources of relationship strain (Apostolou & Wang, 2020, 2021). The current literature supports this prediction.

More specifically, one study employed open-ended questionnaires and in-depth interviews, and identified 78 difficulties that people faced in keeping their intimate relationships (Apostolou & Wang, 2020). Subsequently, the authors asked a sample of 1,099 Greek-speaking participants how likely each difficulty was to cause them strain in an intimate relationship. By applying factor analysis, they classified these difficulties into 12 broader factors, the most important one being “Fading away enthusiasm,” followed by “Long work hours” and “Lack of personal time and space.” One issue with this study was that it asked participants to rate hypothetically how much strain each factor would cause them, and not how each one actually caused them. To address this limitation, a subsequent study asked 1,403 participants from China and Greece who were in an intimate relationship to indicate their agreement on how each of the 78 items caused them strain in their relationship (Apostolou & Wang, 2021). Using principal components analysis, they classified these difficulties into 13 broader factors. Participants rated “Clinginess” as the most important source of relationship strain, followed by “Long work hours,” “Lack of personal time and space,” and “Not making compromises.” In the middle of the hierarchy were “Character issues,” “Lack of effort,” “Violence and addictions,” “Fading away enthusiasm,” and “Bad sex.” At the bottom of the hierarchy were “Infidelity,” “Children,” “Social circle issues,” and “Not monogamous.”

### Current Literature

In our theoretical framework, relationship strain arises predominantly from lack of commitment and support, exhibiting undesirable traits, and constraining a partner. Accordingly, we hypothesize that, in order to improve their intimate relationships, people would employ strategies that aim specifically to address these issues. That is, these strategies would, to some degree, aim to address the sources of strain identified by previous research. In particular, we hypothesize that they would include, giving partners more space, and demonstrating to them that one is committed to the relationship, is supporting, and has desirable traits. The current literature provides support for these hypotheses.

Buss (1988) identified 104 relationship retention acts, and conceptually classified them into 19 broader retention strategies (see also Buss & Shackelford, 1997). Many strategies such as “Derogation of competitors,” “Vigilance,” and “Violence” aimed to produce costs to the intimate partner. On the other hand, he found several strategies aiming to provide benefits to the intimate partner. These strategies included “Sexual inducements” (e.g., He gave in to her sexual requests), “Enhancing physical appearance” (e.g., He made up his face look nice), “Emphasize love and caring” (e.g., He told her that he loved her), and “Submission and debasement” (e.g., He told her that he would change in order to please her).

There have been several other studies on the strategies that people use in order to maintain their intimate relationships (for an inclusive review see Ogolsky et al., 2017). These strategies either aim to keep a relationship at a given state or to improve it (Dindia, 2003). With respect to the former, Ayres (1983) identified three strategies that people use in order to keep their interpersonal relationships stable. Similarly, Dindia and Baxter (1987) examined the acts that couples did in order to maintain or repair their relationship, and conceptually classified them into 12 strategies. Moving on to relationship enhancement strategies, Stafford and Canary (1991) generated several acts that people do in order to maintain a satisfactory relationship through a literature review and by analyzing responses to an open-ended questionnaire taken by married and dating couples. By employing principal components analysis on a sample of 956 participants, they classified these items into five broader factors, namely positivity, openness, assurances, sharing tasks, and social networks. The positivity factor involved acts that aimed to have a more pleasant interaction with one's partner (e.g., acts cheerful and positive when with me). The openness factor involved discussing the quality of the relationship (e.g., tells me how she/he feels about the relationship), and the assurances factor involved convincing the partner that one was committed to the relationship, is faithful, and the relationship has a future. The social networks, involved spend time with friends and family, while the sharing tasks involved equity in task that the couple faced such as household chores. Ogolsky and Bowers (2013), examined different studies and offered meta-analytic evidence which indicated strong associations between these factors and relationship quality (see also Ogolsky et al., 2017).

In the same vein, one study examined romantic partners and adolescent opposite-sex friends during interactions that elicited love and threatened the bond (Gonzaga et al., 2001). They found that providing evidence for a nonverbal display of love correlated with self-reports and partner estimates of love. In addition, the experience and display of love correlated with commitment-enhancing processes (e.g., constructive conflict resolution, perceived trust) when the relationship was threatened. Furthermore, Komiya et al. (2019) argued that periodical gift exchanges works as commitment signals among married couples. In particular, they hypothesized that in a context where there is higher relationship mobility (i.e., people can more easily change intimate partners), there is a higher need for couples to demonstrate their commitment. Consistent with this argument, they found that Japanese married couples who had more relational opportunities more frequently engaged in gift exchanges than those who did not (see also Powell & Van Vugt, 2003; Yamaguchi et al., 2015).

### The Current Study

Given the complexity of the phenomenon and the sensitivity of the factor analysis to the sample used and the items employed, further replication studies are necessary in order to identify the structure of the strategies people use in order to improve their intimate relationships. That is, the current literature may not be inclusive of the relationship enhancement strategies people use, which could be one of the reasons why some predicted strategies, such as giving partners more space, were not reported. Moreover, with the exception of Buss (1988) who focused, however, on broader mate retention strategies, previous research in the area did not attempt to understand the evolutionary logic of the relationship enhancement strategies people use. Accordingly, the current research had three main objectives: (a) to place relationship enhancement strategies into an evolutionary perspective; (b) to identify the strategies that people employ in order to improve relationships with their partners in the Greek cultural context (Studies 1 and 2), and to test the hypothesis that the produced factors would include strategies that aim to give partners more space, demonstrating to them that one is committed to the relationship, supporting, and has desirable traits (Study 2); (c) to assess the effectiveness of the identified strategies on improving relationships (Study 3); (d) to identify the presence of sex and age differences in the willingness to use and the effectiveness of each strategy, without making, however, directional hypotheses.

## Study 1

### Methods

#### Participants

The research project was designed at a university located in the Republic of Cyprus, and run online. The study received ethics approval from the university's ethics review board. Participants were recruited by forwarding the link of the study to students in various departments, asking them to forward it further, and by promoting it on Facebook and Instagram to participants living in Greece and in the Republic of Cyprus. The initial sample consisted of 281 participants. However, in order to increase validity, we excluded participants’ answers who had no relationship experience; that is, they indicated that they were currently single and they had never been in a relationship before. Accordingly, we employed a sample of 219 Greek-speaking participants (121 women, and 98 men) who had relationship experience. The mean age of women was 32.38 years (*SD* = 12.85), and the mean age of men was 36.96 years (*SD* = 12.75). In addition, 38.1% of the participants indicated that they were in a relationship, 29.3% married, 25% single, and 7.6% indicated their marital status as other.

#### Materials

The open-ended questionnaire had two parts. In the first part, participants were asked the following: “Write down some things you have done in the past or you are likely to do in the future in order to improve your relationship with your partner.” In the second part, demographic information was collected, including sex, age, marital status, and whether one had been in an intimate relationship in the past.

#### Data Analysis and Results

The data were analyzed independently by one of the authors and a graduate student using the following procedure: Each researcher went through the responses from the open-ended questionnaires, and prepared a list of acts that participants indicated that they had or would perform in order to improve their intimate relationships. Acts with vague wording or confusing meaning were eliminated. After processing the data, the researchers compared their respective lists. In case of discrepancies, some of the data were reexamined, and the other authors were also consulted. The researchers finally agreed to a list of 81 acts such as “I would try to understand more of his/her needs,” “I would try to create new experiences together,” and “I would make more compromises” that people would use in order to improve their intimate relationships. All the acts were listed in Table 1.

**Table 1.** The Items Identified in Study 1 and the Extracted Strategies in Study 2.

Factors Items	Factor loadings	Cronbach's α
Try to understand her/his needs		.91
I would try to understand his/her needs more	.557	
I would try harder to understand how he/she thinks	.494	
I would try to understand more of his/her wishes	.476	
I would try to listen to him/her more	.465	
I would try to understand him/her more	.437	
I would listen more to his/her problems	.396	
I would try to improve our communication	.387	
I would try to get into his/her shoes	.381	
I would show him/her my understanding	.281	
I would try to respect him/her more	.280	
I would try to help him/her deal more with his/her problems	.274	
		
Improve looks		.78
I would improve my looks	.721	
I would renew my wardrobe	.713	
I would make him/her a little jealous	.651	
I would lose weight	.615	
I would give him/her gifts	.415	
		
Create experiences together		.84
I would suggest we go on a trip	.778	
I would organize an excursion	.635	
I would try to create new experiences together	.600	
I would try to renew our relationship (e.g., through activities we both enjoy)	.467	
I would be more involved in things he/she likes	.442	
We would go out on a romantic date	.422	
I would make him/her surprises	.392	
I would try to avoid as much as possible the routine in our relationship	.384	
		
Self-improvement		.76
I would attend seminars on improving relationships	.844	
I would go to a psychologist/psychiatrist/relationship counselor to help me improve my attitude toward my partner	.831	
I would read books on improving relationships	.765	
I would make efforts for self-improvement	.362	
		
Give my partner more space		.79
I would give him/her more freedom	.792	
I would give him/her more time and space to be with his/her friends	.751	
I would give him/her more space	.704	
I would support his/her choices	.407	
I would be less pushy	.403	
I would show more recognition for what he/she does	.324	
		
Be less withdrawn and grumpy		.54
I would be less withdrawn	−.676	
I would try not to pass on my problems to him/her	−.517	
I would be less grumpy	−.474	
		
Show more interest in my partner		.85
I would dedicate more time to him/her	−.546	
I would try to spend more time together	−.472	
I would put him/her in a higher priority	−.468	
I would criticize him/her less	−.427	
I would show more interest in him/her	−.419	
I would show him/her more trust	−.383	
I would try to be more there for him/her	−.322	
I would take better care of him/her	−.308	
		
Discuss our problems together		.82
I would talk to him/her about anything that bothers me	.809	
I would discuss our problems more	.651	
I would talk to him/her about anything that bothers him/her	.564	
I would try to talk more in order to avoid misunderstandings	.463	
I would discuss any problems that arise	.454	
		
Show her/him my love		.79
I would show him/her my love	.468	
I would tell him/her more frequently “I love you”	.463	
I would try to improve our sex life	.392	
I would make more effort to accept him/her as he/she is	.332	
I would express my feelings more	.297	
I would try to apologize more often when I do something wrong	.263	
I would claim him/her more	.249	
		
Look after myself		.48
I would try to look more after myself in order to feel good about me	.703	
I would set limits on the things I want or am willing to do in a relationship	.604	
		
Open to her/his criticism		.41
I would try to be more receptive to his/her criticism	.475	
I would try to find what is wrong with our relationship and fix it	.341	
		
Make concessions and compromises		.89
I would be more conciliatory	−.639	
I would make more compromises	−.635	
I would be more patient	−.577	
I would try to tolerate his/her quirks more	−.568	
I would be more tolerant of his/her demands	−.414	
I would do him/her more favors	−.409	
I would try to respond more to his/her wishes	−.397	
I would fulfill his/her desires in sex	−.353	
I would make more effort to accept his/her idiosyncrasies	−.332	
I would try to meet his/her needs more	−.313	
I would try to change things that bother him/her	−.250	
		
Build trust		.54
I would build more trust, for example, by showing that I do not mind he/she seeing my cell phone because I have nothing to hide	.498	
I would try to be more honest with him/her	.477	
I would try harder to keep the agreements we have made between us	.350	
		
Exercise self-control		.77
I would try to react calmly when he does or says something that bothers me	−.641	
I would try to change some of my bad habits	−.436	
I would try not to react straightaway when he does or says something that bothers me	−.433	
I would try to have more self-control over my anger/irritability	−.416	
I would try to be less selfish	−.395	
I would become less demanding	−.295	

## Study 2

### Methods

#### Participants

In order to recruit participants, we employed the same procedure as in Study 1. The initial sample included 663 participants; however, similar to Study 1, in order to increase validity, we excluded participants who indicated that they were currently single, and that they never had an intimate relationship before. Accordingly, the analysis was performed on a sample of 511 Greek-speaking participants (309 women, 198 men, and four participants who did not report their sex) who indicated that they had relationship experience. The mean age of women was 33.75 years (*SD* = 1.69), and the mean age of men was 36.23 years (*SD* = 13.38). Moreover, 36% of the participants indicated that they were in a relationship, 30.1% married, 29% single, and 4.9% indicated their marital status as other.

#### Materials

The survey consisted of two parts. In the first part, participants were given the following scenario: “Suppose you are in a romantic relationship. Indicate to what extent you would perform each of the following acts in order to improve your relationship with your partner,” and subsequently, they were given the 81 acts identified in Study 1 to rate in the following scale: 1 (*not at all likely to do it*), 5 (*very likely to do it*). The order of presentation of each act was randomized across participants. In the second part, participants were asked about their demographic information, including their sex, age, marital status, and whether they had been in an intimate relationship before.

#### Data Analysis

In order to classify acts to broader strategies, we performed principal components analysis using the direct oblimin as the rotation method. In order to identify significant sex, age, and marital status effects, we performed a series of MANCOVA tests. More specifically, the acts composing each identified strategy were entered as dependent variables, sex, and marital status were entered as categorical independent variables, and age was entered as a continuous independent variable. The analysis was performed separately for each identified strategy.

### Results

The KMO statistic was .952, indicating that our sample was very good for principal components analysis to be performed. Using the Keiser criterion (Eigenvalue >1), we extracted 14 distinct strategies, which are presented in Table 1. The Cronbach's alpha for each strategy ranged from .41 to .91. Factors consisting of two or three items usually have low alphas, without this indicating a low internal consistency (Tavakol & Dennick, 2011).

The first strategy to emerge was the “Try to understand her/his needs,” where people would attempt to improve their intimate relationships by trying to better understand their partners' needs. One way they could achieve this goal was to listen more to their partners and to their problems. The second factor to emerge was the “Improve looks,” where participants would improve their appearance, by renewing their wardrobe and losing weight. Such changes in looks would also aim to make partners jealous. People would also attempt to “Create experiences together” with their partners, by organizing trips and excursions, and going out on romantic dates. Moreover, in order to improve their relationships, participants would make attempts for “Self-improvement” by attending seminars, getting help from specialists, and reading relevant books. In the “Give my partner more space” strategy, people would attempt to improve their relationship by giving their partners more freedom, more time to be with their friends, and by trying to be less pushy. They would also try to “Be less withdrawn and grumpy.” In the “Show more interest in my partner” strategy, people would put their partners in higher priority and would show them more interest and trust.

Moving on, in the “Discuss our problems together” strategy, people would attempt to improve their relationship by discussing any problems that arise with their partners. In the “Show her/him my love” strategy, people would try to demonstrate their love to their partners by telling them more frequently that they loved them, improving their sexual life, and claiming them more. In addition, in the “Look after myself” strategy, people would attempt to be better-off with themselves as a way to be better-off with their partners. In the “Open to her/his criticism” strategy, people would be more open to their partners' criticism as a way to figure out what was wrong with the relationship. People would also “Make concessions and compromises,” and they would further try to be more patient and accepting of their partners' idiosyncrasies. They would also try to “Build trust” with their partners, by being honest with them, and by keeping the agreements they have made with them. Moreover, people would attempt to “Exercise self-control,” reacting calmly when their partners would do or say something that bothered them.

In order to identify which strategies people were more likely to use in order to improve their intimate relationships, we estimated the means and the standard deviations for each one. As we can see from Table 2, at the top of the hierarchy, was the “Try to understand her/his needs,” followed by the “Discuss our problems together” and the “Show more interest in my partner.” The least likely to be used strategies were the “Improve looks,” the “Self-improvement,” and the “Make concessions and compromises.”

**Table 2.** Mean Scores, Sex, Age, and Marital Status Effects in Study 2.

Strategies	Overall	Sex		Age		Marital status	
	*M* (*SD*)	*p*	η*_p_*^2^	*p*	η*_p_*^2^	*p*	η*_p_*^2^
Try to understand her/his needs	4.42 (0.53)	.414	.025	(−)<.001	.072	.248	.028
Discuss our problems together	4.41 (0.58)	.018	.029	.011	.032	.458	.011
Show more interest in my partner	4.24 (0.58)	.717	.012	.023	.039	.015	.030
Create experiences together	4.23 (0.63)	.450	.017	.011	.044	.045	.017
Build trust	4.22 (0.69)	.053	.017	.310	.008	.288	.008
Show her/him my love	4.18 (0.61)	.035	.033	(−)<.001	.058	.938	.009
Give my partner more space	4.17 (0.60)	.617	.010	.005	.040	.617	.011
Open to her/his criticism	4.0782	.007	.022	.067	.012	.367	.007
Exercise self-control	3.9253	.563	.011	.100	.023	.400	.014
Look after myself	3.8730	.185	.007	.601	.002	.118	.011
Be less withdrawn and grumpy	3.7925	.619	.004	.093	.014	.854	.003
Make concessions and compromises	3.7878	<.001	.086	.313	.028	.715	.021
Self-improvement	3.3092	.770	.004	.159	.014	.727	.006
Improve looks	3.1308	.185	.016	.023	.028	.557	.010

*Note.* The signs in parenthesis indicate the direction of the relationship.

We performed 14 MANCOVA tests, one for each identified strategy. In order to avoid the problem of alpha inflation arising from multiple comparisons, Bonferroni correction could be applied, setting the alpha level to .004 (.05/14). A significant main effect of sex was found only for the “Make concessions and compromises” strategy, where men gave higher scores (*M* = 3.95, *SD* = 0.61) than women (*M* = 3.69, *SD* = 0.68). A significant main effect of age was found for the “Try to understand her/his needs” and the “Show her/him my love” strategies. In both cases, the regression coefficient was negative, indicating that older participants gave lower scores than younger participants. In addition, marital status was not significant for any of the identified strategies.

We have also attempted to examine whether the identified strategies could be classified into even broader domains. For this purpose, we performed second-order principal components analysis on the 14 variables depicting the mean scores for each strategy, using the direct oblimin as the rotation method. Based on the Keiser criterion (Eigenvalue >1), we extracted two domains. As we can see from Table 3, the first domain reflected strategies which were focused on partner, while the second domain reflected strategies which were focused on self.

**Table 3.** The Extracted Domains in Study 2.

Domains Factors	Factor loadings
Focus on partner	
Try to understand her/his needs	.947
Show more interest in my partner	.901
Show her/him my love	.800
Discuss our problems together	.781
Build trust	.778
Give my partner more space	.765
Exercise self-control	.721
Open to her/his criticism	.693
Make concessions and compromises	.673
Create experiences together	.670
Be less withdrawn and grumpy	.596
	
Focus on self	
Self-improvement	.774
Improve looks	.739
Look after myself	.642

## Study 3

### Methods

#### Participants

In order to recruit participants, we employed the same method as in Study 1. The initial sample consisted of 426 participants; however, similar to Studies 1 and 2, we excluded participants who indicated that they were currently single and they had never been in a relationship before. Accordingly, the analysis was performed on a sample of 367 Greek-speaking participants (206 women, 160 men, and one participant who did not indicate her/his sex). The mean age of women was 28.89 years (*SD* = 7.58), and the mean age of men was 29.04 years (*SD* = 5.73). Moreover, 48.9% of the participants indicated that they were in a relationship, 29.5% single, 17.2% married, and 4.4% indicated their marital status as other.

#### Materials

The survey had two parts. In the first part, participants were given the following scenario: “Suppose you are in a romantic relationship. Below you will find a series of acts that your partner is likely to perform. Please indicate how effective you think each one would be in improving your relationship with her/him”: Subsequently, participants were given the 81 acts identified in Study 1 to rate on the following scale: 1 (*not at all effective*), 5 (*very effective*). The order of presentation of the different acts was randomized across participants. In the second part, participants indicated their demographic information, including their sex, age, marital status, and whether they had been in an intimate relationship in the past.

#### Data Analysis

In order to identify significant sex, age, and marital status effects, we performed a series of MANCOVA tests. More specifically, the acts composing each strategy identified in Study 2 were entered as dependent variables, sex, and marital status were entered as categorical independent variables, and age was entered as a continuous independent variable. The analysis was performed 14 times, once for each identified strategy.

### Results

In order to examine which strategy was considered to be more effective, we estimated the means and the standard deviations for each one, and we placed them in a hierarchical order in Table 4. The most effective strategy for improving relationships was rated to be the “Discuss our problems together” followed by the “Try to understand her/his needs” and the “Show her/him my love.” The least effective ones included the “Improve looks,” the “Self-improvement,” and the “Be less withdrawn and grumpy.” Furthermore, in order to estimate whether individuals were more likely to employ the strategies that were rated to be more effective, we correlate the rankings in Study 2 with the rankings in Study 3. Spearman's correlation was strong, *r*(14) = .916, *p* < .001 (two-tailed), indicating that the hierarchy of strategies people was willing to use matched well the hierarchy of how effective these strategies were judged to be.

**Table 4.** Mean Scores, Sex, Age, and Marital Status Effects in Study 3.

Strategies	Overall	Sex		Age		Marital status	
	*M* (*SD*)	*p*	η*_p_*^2^	*p*	η*_p_*^2^	*p*	η*_p_*^2^
Discuss our problems together	4.36 (0.67)	.031	.036	.552	.012	.568	.013
Try to understand her/his needs	4.20 (0.62)	.007	.076	.379	.035	.454	.033
Show her/him my love	4.06 (0.62)	.024	.047	.781	.012	.143	.027
Show more interest in my partner	4.04 (0.67)	<.001	.089	.044	.047	.713	.019
Open to her/his criticism	4.02 (0.78)	.208	.009	.446	.005	.358	.010
Build trust	4.00 (0.77)	.087	.019	.648	.005	.353	.010
Create experiences together	3.97 (0.70)	.072	.042	.340	.027	.262	.027
Give my partner more space	3.94 (0.73)	.079	.033	.146	.028	.991	.007
Exercise self-control	3.94 (0.67)	.108	.030	.031	.040	.496	.017
Make concessions and compromises	3.72 (0.66)	.133	.048	.119	.050	.103	.043
Look after myself	3.60 (0.90)	.197	.010	.027	.021	.086	.016
Be less withdrawn and grumpy	3.54 (0.76)	<.001	.061	.005	.037	.754	.006
Self-improvement	3.01 (0.96)	.039	.029	(+).001	.053	.830	.007
Improve looks	2.44 (0.87)	.013	.042	(+).003	.052	.040	.025

*Note.* The signs in parenthesis indicate the direction of the relationship.

Turning to MANCOVA results, Bonferroni correction could be used as in Study 2, setting alpha to .004. A significant main effect of sex was found for the “Show more interest in my partner” strategy, where women gave higher scores (*M* = 4.24, *SD* = 0.61) than men (*M* = 3.77, *SD* = 0.64), and for the “Be less withdrawn and grumpy,” where women gave also higher scores (*M* = 3.61, *SD* = 0.75) than men (*M* = 3.46, *SD* = 0.76). Moreover, there was a significant main effect of age on the “Self-improvement” and the “Improve looks” strategies, both with a positive regression coefficient, indicating that older participants rated these strategies as more effective than younger participants. In addition, no significant main effects of marital status were detected.

## Discussion

By using a combination of qualitative and quantitative research methods, we identified 81 acts that we subsequently classified into 14 broader strategies that people employ in order to improve their intimate relationships. Trying to understand partners' needs, discussing problems with partners, and showing more interest in them, were among the most likely to be used and the most effective strategies. On the other hand, making efforts for self-improvement, and enhancing one's looks, were the least likely to be used, and were rated as the least effective strategies. Men and women, and people of different ages, were generally in agreement over which strategies they were willing to use, and how effective these strategies would be on them. We have also classified the identified strategies into two broader domains, which indicate that, in their attempt to improve their intimate relationships, people can focus on their partners, and/or on themselves. Nevertheless, the strategies that loaded in the latter domain were rated as not particularly effective.

We have argued that one source of relationship strain is the intimate partner exhibiting lack of commitment to the relationship. For instance, previous research has identified partners' behaviors that indicate lack of commitment, including “Lack of effort,” “Fading away enthusiasm,” “Long work hours,” “Not making compromises,” and “Infidelity” as a frequent source of relationship strain (Apostolou & Wang, 2021). We hypothesized that, in order to improve their intimate relationships, people would employ strategies that would convey to their partners the message that they are committed to the relationship. Consistent with this hypothesis, we have identified a number of strategies, including “Show more interest in my partner,” “Create experiences together,” “Build trust,” “Discuss our problems together,” “Show her/him my love,” and “Make concessions and compromises,” which enable people to indicate to their partners that they are committed to the relationship. The “Discuss our problems together,” the “Show more interest in my partner,” and the “Create experiences together” were among the most likely to be used strategies, while the “Discuss our problems together,” the “Show more interest in my partner,” and the “Show her/him my love” were rated to be the most effective strategies.

We have argued further that, another source of relationship strain, was the partner being controlling. In particular, research in the area has identified “Clinginess” and “Lack of personal time and space” to be among the most frequent sources of relationship strain (Apostolou & Wang, 2021). Accordingly, in order to improve their intimate relationships, people employ the “Give my partner more space,” reducing in effect the control they exercise over their partners. Nevertheless, participants indicated that this strategy was among the least likely to be used, one reason being that it was not particularly effective. Another reason can be that, by giving more space to their partners, people expose themselves to the risk of infidelity.

We hypothesized further that lacking desirable qualities was another source of relationship strain. Previous research has identified “Character issues” and “Lack of effort” to be frequently reported sources of relationship strain (Apostolou & Wang, 2020). Accordingly, we hypothesized that people would use strategies that enable them to demonstrate to their partners that they have desirable traits or at least that they do not have undesirable ones. One such trait is good looks (Buss, 2016), so people use the “Improve looks” strategy to enhance their appearance. The “I would make him/her a little jealous” act loaded here, suggesting that this strategy aimed further to trigger some insecurity to partners that they may lose their mates to others. Such insecurity could strengthen the relationship, but it may also have the opposite effect, triggering bursts of jealousy that could negatively affect the relationship, which could be one of the reasons why it was rated as the least likely to be used and the least effective strategy. The most valued traits in a partner related to character, including kindness and understanding, and pleasant personality (Buss & Schmitt, 2019). Accordingly, in the “Try to understand her/his needs,” people indicate that they are understanding to their partners' needs, and in the “Make concessions and compromises” that they are flexible. The former strategy was rated as the second most effective one in improving an intimate relationship. Similarly, in the “Be less withdrawn and grumpy” and in the “Exercise self-control” strategies, people attempt not to convey to their partners the message that they are unpleasant, aggressive or that they lack self-control.

The two sexes appeared to be in agreement over their willingness to employ the identified strategies, with the only significant sex difference being over the “Make concessions and compromises” strategy, where men indicated a higher willingness to use it than women.

Similarly, the two sexes were in agreement over how effective they considered each strategy to be. Significant sex differences were found only for the “Show more interest in my partner” and the “Be less withdrawn and grumpy,” which were rated as more effective by women. We do not have a working hypothesis for the existence of these sex differences, and future research needs to attempt to replicate our results in different samples.

Although there is overlap, the factor structure we have identified here is considerably different than the one Stafford and Canary (1991) have produced. More specifically, the latter study has identified five such strategies, and in the current research, we have identified 14 such strategies. One reason for this difference is that we have employed a larger sample of participants in Study 1 in order to identify the specific acts that people perform in order to improve their intimate relationships, so we had a more inclusive list. Cultural differences and time of the study (Stafford and Canary's study took place more than 30 years ago) may also be at play. More replication studies are needed for the factor structure of relationship-enhancing strategies to be understood.

Our findings have potential practical value for people aiming to improve their intimate relationships, and for mental health professionals wanting to help clients who face difficulties in their intimate relationships. This is even more so, if one considers that intimate relationships appear to be in crisis, with singlehood and divorce being on the rise (Cherlin, 2009; Klinenberg, 2013). In particular, aiming to discuss problem that arises with a partner, show one's interest to a partner, and create experiences with a partner, are relatively easy to do (and possibly enjoyable in the case of creating new experiences), and as our results indicate, can also be very effective in reducing relationship strain. On the other hand, strategies such as improving one's looks and becoming less distant would probably have limited effect on improving an intimate relationship. In addition, a combination of the identified strategies is likely to have stronger effect than employing each one individually. For instance, people discussing problems with their partners, but also showing them their love and interest, and creating new experiences together, could greatly improve one's intimate relationship. Future research needs to investigate further the combined effects of the identified strategies on relationship quality.

One limitation of the current research is that it employed non-probability samples, so our findings may not readily generalize to the population. Furthermore, we used self-report instruments, which have several biases, including participants not being able to accurately assess how likely they were to use each strategy, and how effective each strategy would be on them. In addition, our research was confined to the Greek cultural context, and its findings may not generalize to different cultural settings. Accordingly, cross-cultural research is necessary in order to examine how cultural factors affect the identified strategies. Moreover, the mean scores for most of the identified strategies were high, indicating a high willingness to use the identified strategies for the purpose of improving relationships. However, the current research is not informative on the degree that this willingness materializes in actual use of these strategies. The high rates of divorce and relationship failures suggest that people may not use these strategies as frequently as their willingness to do so suggests. Accordingly, future research should attempt to examine the degree that people actually use these strategies, as well as the factors which predict whether they would do so, such a relationship satisfaction and having children.

Intimate relationships constitute a fascinating aspect of human behavior, and in the current research, we have identified 14 strategies that people employ in order to improve them. We have also found that the two sexes and people belonging to different age groups were generally in agreement over which strategies they would use, and how effective they would be on them. The complexity of the phenomenon requires considerable more theoretical and empirical work if relationship enhancement strategies are to be better understood.

## References

[bibr1-14747049221147154] Albert G. Arnocky S. (2016). Use of mate retention strategies. In Weekes-Shackelford V. Shackelford T. (Eds.), Encyclopedia of evolutionary psychological science. Springer.

[bibr2-14747049221147154] Apostolou M. (2021). What makes it difficult to keep an intimate relationship: Evidence from Greece and China. Evolutionary Psychology, 19(1). 10.1177/1474704920987807 PMC10358403 33525960

[bibr3-14747049221147154] Apostolou M. (2022). Why people keep an intimate relationship: Investigating ultimate and proximate reasons. Human Nature, 33(1), 62–81. 10.1007/s12110-021-09421-9 35037235

[bibr4-14747049221147154] Apostolou M. Wang Y. (2019). The association between mating performance, marital status, and the length of singlehood: Evidence from Greece and China. Evolutionary Psychology, 17(4), 147470491988770. 10.1177/1474704919887706 PMC10480974 31722550

[bibr5-14747049221147154] Apostolou M. Wang Y. (2020). The challenges of keeping an intimate relationship: An evolutionary examination. Evolutionary Psychology, 18(3). 10.1177/1474704920953526 PMC10358406 32885673

[bibr6-14747049221147154] Ayres J. (1983). Strategies to maintain relationships: Their identification and perceived usage. Communication Quarterly, 31(1), 62–67. 10.1080/01463378309369487

[bibr7-14747049221147154] Baumeister R. F. Leary M. R. (1995). The need to belong: Desire for interpersonal attachments as a fundamental human motivation. Psychological Bulletin, 117(3), 497–529. 10.1037/0033-2909.117.3.497 7777651

[bibr8-14747049221147154] Buss D. M. (1988). From vigilance to violence: Tactics of mate retention in American undergraduates. Ethology and Sociobiology, 9(5), 291–317. 10.1016/0162-3095(88)90010-6

[bibr9-14747049221147154] Buss D. M. (2000). The dangerous passion. Bloomsbury.

[bibr10-14747049221147154] Buss D. M. (2016). The evolution of desire: Strategies of human mating (4th ed.). Basic Books.

[bibr11-14747049221147154] Buss D. M. Goetz C. Duntley J. D. Asao K. Conroy-Beam D. (2017). The mate switching hypothesis. Personality and Individual Differences, 104, 143–149. 10.1016/j.paid.2016.07.022

[bibr12-14747049221147154] Buss D. M. Schmitt D. P. (1993). Sexual strategies theory: An evolutionary perspective on human mating. Psychological Review, 100(2), 204–232. 10.1037/0033-295X.100.2.204 8483982

[bibr13-14747049221147154] Buss D. M. Schmitt D. P. (2019). Mate preferences and their behavioral manifestations. Annual Review of Psychology, 70, 77–110. 10.1146/annurev-psych-010418-103408 30230999

[bibr14-14747049221147154] Buss D. M. Shackelford T. K. (1997). From vigilance to violence: Mate retention tactics in married couples. Journal of Personality and Social Psychology, 72(2), 346–361. 10.1037/0022-3514.72.2.346 9107005

[bibr15-14747049221147154] Cherlin A. J. (2009). The marriage-go-round. Alfred A. Knopf.

[bibr16-14747049221147154] Dindia K. (2003). Definitions and perspectives on relational maintenance communication. In Canary D. J. Dainton M. (Eds.), Maintaining relationships through communication (pp. 1–28). Lawrence Erlbaum Associates.

[bibr17-14747049221147154] Dindia K. Baxter L. A. (1987). Strategies for maintaining and repairing marital relationships. Journal of Social and Personal Relationships, 4(2), 143–158. 10.1177/0265407587042003

[bibr18-14747049221147154] Gangestad S. W. Simpson J. A. (2000). The evolution of human mating: Trade-offs and strategic pluralism. Behavioral and Brain Sciences, 23(4), 573–587. 10.1017/S0140525X0000337X 11301543

[bibr19-14747049221147154] Gonzaga G. C. Keltner D. Londahl E. A. Smith M. D. (2001). Love and commitment problem in romantic relations and friendship. Journal of Personality and Social Psychology, 81(2), 247–262. 10.I037//0022-3514.81.2.247 11519930

[bibr20-14747049221147154] Hawkes K. O’Connell J. F. Blurton Jones N. G. (1989). Hardworking Hadza grandmothers. In Standen V. Foley R. A. (Eds.), Comparative socioecology: The behavioural ecology of humans and other mammals (pp. 341–366). Blackwell Scientific.

[bibr21-14747049221147154] Kim P. S. James E. Coxworth J. E. Hawkes K. (2012). Increased longevity evolves from grandmothering. Proceedings of the Royal Society B: Biological Sciences, 279(1749), 4880–4884. 10.1098/rspb.2012.1751 PMC3497232 23097518

[bibr22-14747049221147154] Klinenberg E. (2013). Going solo: The extraordinary rise and surprising appeal of living alone. Duckworth Overlook.

[bibr23-14747049221147154] Komiya A. Ohtsubo Y. Nakanishi D. Oishi S. (2019). Gift-giving in romantic couples serves as a commitment signal: Relational mobility is associated with more frequent gift-giving. Evolution and Human Behavior, 40(2), 160–166. 10.1016/j.evolhumbehav.2018.10.003

[bibr24-14747049221147154] Lancaster J. B. Lancaster C. S. (1987). The watershed: Change in parental investment and family-formation strategies in the course of human evolution. In Lancaster J. B. Altmann J. Rossi A. S. Sherrod L. R. (Eds.), Parenting across the life span: Biosocial dimensions (pp. 187–205). Aldine Publishing.

[bibr25-14747049221147154] Nesse R. M. (2005). Natural selection and the regulation of defenses. Evolution and Human Behavior, 26(1), 88–105. 10.1016/j.evolhumbehav.2004.08.002

[bibr26-14747049221147154] Nesse R. M. (2018). The smoke detector principle. Evolution, Medicine, and Public Health, 2019(1), 1. 10.1093/emph/eoy034 30697424 PMC6343816

[bibr27-14747049221147154] Ogolsky B. Bowers J. (2013). A meta-analytic review of relationship maintenance and its correlates. Journal of Social and Personal Relationships, 30(3), 343–367. 10.1177/0265407512463338

[bibr28-14747049221147154] Ogolsky B. G. Monk J. K. Rice T. M. Theisen J. C. Maniotes C. R. (2017). Relationship maintenance: A review of research on romantic relationships. Journal of Family Theory & Review, 9(3), 275–306. 10.1111/jftr.12205

[bibr29-14747049221147154] Powell C. Van Vugt M. (2003). Genuine giving or selfish sacrifice? The role of commitment and cost level upon willingness to sacrifice. European Journal of Social Psychology, 33(3), 403–412. 10.1002/ejsp.154

[bibr30-14747049221147154] Raley R. K. Bumpass L. L. (2003). The topography of the divorce plateau: Levels and trends in union stability in the United States after 1980. Demographic Research, 8, 245–260. 10.4054/DemRes.2003.8.8

[bibr31-14747049221147154] Stafford L. Canary D. J. (1991). Maintenance strategies and romantic relationship type, gender and relational characteristics. Journal of Social and Personal Relationships, 8(2), 217–242. 10.1177/0265407591082004

[bibr32-14747049221147154] Tavakol M. Dennick R. (2011). Making sense of Cronbach’s alpha. International Journal of Medical Education, 2(6), 53–55. 10.1016/S0092-6566(02)00505-6 28029643 PMC4205511

[bibr33-14747049221147154] Thomas A. G. Jonason P. K. Blackburn J. Kennair L. E. O. Lowe R. Malouff J. Li N. P. (2020). Mate preference priorities in the east and west: Across–cultural test of the mate preference priority model. Journal of Personality, 88(3), 606–620. 10.1111/jopy.12514 31494937

[bibr34-14747049221147154] Tooby J. Cosmides L. (2015). The theoretical foundations of evolutionary psychology. In Buss D. M. (Ed.), The handbook of evolutionary psychology: Vol. 1. Foundations (pp. 3–87). Wiley.

[bibr35-14747049221147154] Walter K. V. Conroy-Beam D. Buss D. M. Asao K. Sorokowska A. Sorokowski P. Zupančič M. (2020). Sex differences in mate preferences across 45 countries: A large-scale replication. Psychological Science, 31(4), 408–423. 10.1177/0956797620904154 32196435

[bibr36-14747049221147154] Yamaguchi M. Smith A. Ohtsubo Y. (2015). Commitment signals in friendship and romantic relationships. Evolution and Human Behavior, 36(6), 467–474. 10.1016/j.evolhumbehav.2015.05.002

